# Vertebral artery dissection presenting as a Brown-Séquard syndrome: a case report

**DOI:** 10.1186/1752-1947-3-107

**Published:** 2009-11-04

**Authors:** Saul Miller, Dan Kottachchi, Eli Miller

**Affiliations:** 1 McMaster University, Department of Internal Medicine, Hamilton, Ontario, Canada

## Abstract

**Introduction:**

Vertebral artery dissection has become increasingly recognized as an important cause of stroke. It usually presents with posterior headache or neck pain followed within hours or days by signs of posterior circulation stroke. To the best of our knowledge, the clinical presentation of a Brown-Séquard syndrome with a vertebral artery dissection has been reported only once before.

**Case presentation:**

An otherwise healthy 35-year-old man presented with acute left-sided weakness. He had experienced left-sided posterior neck pain after a 4-hour flight 4 weeks previously. Physical examination was consistent with a left Brown-Séquard syndrome. Magnetic resonance angiography showed evidence of left vertebral artery dissection. He improved after therapy with anticoagulants.

**Conclusion:**

We report a case of an unusual presentation of a relatively uncommon condition. This diagnosis should be considered early in relatively young patients with stroke-like symptoms or unexplained neck pain, because missing a dissection can result in adverse outcomes.

## Introduction

Vertebral artery dissection (VAD) usually presents with posterior headache or neck pain followed within hours or days by posterior circulation stroke. Rarely, the dissection may present with signs or symptoms referable only to the cervical spinal cord. We report a case of VAD presenting as a Brown-Séquard syndrome, which, to the best of our knowledge, has been reported only once before.

## Case presentation

A 35-year-old right-handed man who worked in a bank was admitted to hospital with complaints of neck pain and left-sided weakness. The patient had developed a sharp pain in the left side posterior aspect of his neck and occiput after a 4-hour flight, 4 weeks before presentation. At the time he felt nauseated but did not vomit, and he was treated conservatively with ultrasound and massage. The weakness began acutely after he had turned his head to one side and rapidly progressed from his left leg to his left arm. He was immediately unable to walk and subsequently began to experience tingling sensations in his right arm and leg.

There was no history of head trauma or neck manipulation, no past medical or surgical history, and no previous use of medications or previous infectious symptoms. The patient was a non-smoker, did not have diabetes and was normotensive. There was no family history of thrombophilia or venous throboembolism. Both his father and uncle had suffered from ischemic strokes in their late forties. He was born in the Philippines and had lived in Canada since early childhood.

On examination, level of consciousness, cognition and speech were normal. His blood pressure was 144/96 mmHg. The cranial nerve examination was normal. There was Medical Research Council (MRC) grade 4 out of 5 weakness affecting the left arm and left leg, with active movement possible against gravity and minimal resistance. Pinprick and temperature sensation were diminished on the right side from the second cervical vertebra (C2). Vibration and joint position were diminished on the left side below C2. The left plantar reflex was equivocal and the right was flexor. The remainder of the physical examination was normal. The results of the examination were consistent with a Brown-Séquard syndrome on the left at the level of C1-C2.

Hematologic, biochemical and immunologic investigations were normal and electrocardiogram and chest radiography were unremarkable. The lumbar puncture cerebrospinal fluid (CSF) protein was 0.22 g/l and glucose was 4.9 mmol/l (blood glucose 8.8 mmol/l). There was one white cell, two red cells, no oligoclonal banding and no xanthrochromia in 10 ml of CSF.

**Figure 1:**
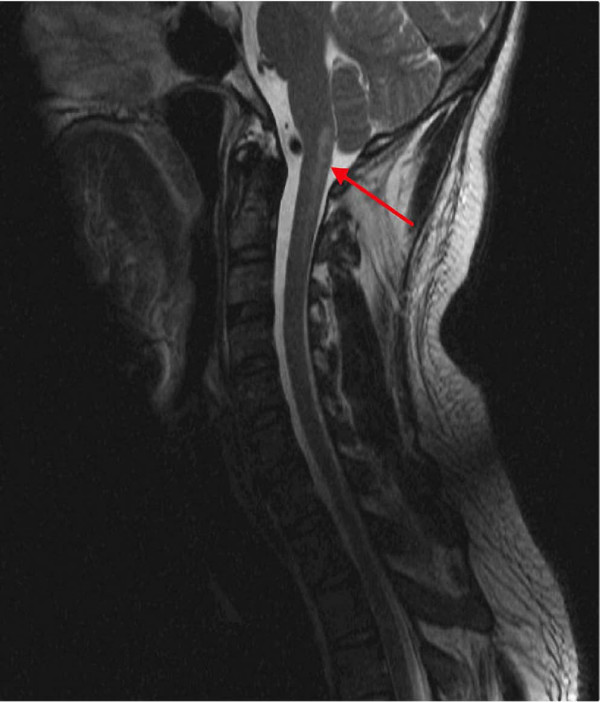
**Sagittal T2 restore magnetic resonance image demonstrating a posterior hyperintense lesion (red arrow) at the cranio-cervical junction involving the caudal medulla and the upper cervical cord**.

**Figure 2:**
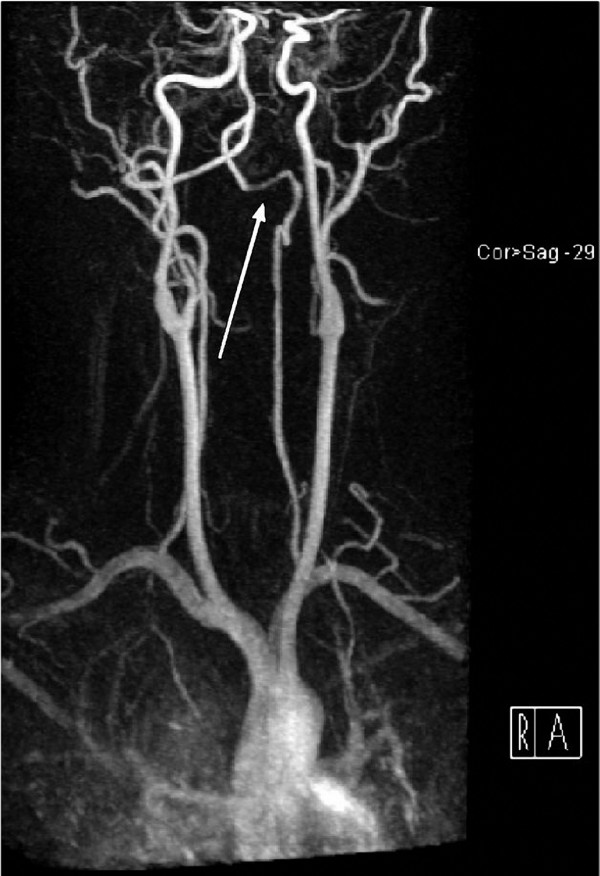
**Magnetic resonance imaging maximum-intensity projection with gadolinium demonstrates irregularity and decreased vessel caliber to the V3 segment of the left vertebral artery (white arrow)**. This is a characteristic finding in an arterial dissection.

T2-weighted magnetic resonance (MR) imaging performed 2 days after presentation revealed a hyperintense signal associated with restrictive diffusion from the left inferior medulla and obex to the level of C2-C3, consistent with infarction (Figure 1). Three-dimensional time-of-flight MR angiography showed narrowing of the left vertebral artery over the arch of C1 with a high signal parallel to the narrowed lumen, consistent with intramural hematoma and dissection (Figure 2).

The patient was started on anticoagulation therapy with heparin and bridged to warfarin as secondary prevention. When the patient was discharged one month later, he was doing well, with improving, although not yet normal, strength in his left arm and leg.

## Discussion

Cervical artery dissection has become increasingly recognized as an important cause of posterior circulation stroke in the young [1]. VAD typically presents with ipsilateral neck or occipital pain preceding a posterior circulation ischemic event. A history of a delay between the onset of headache or posterior neck pain and subsequent neurological deficits should raise the suspicion of VAD. Delays as long as 6 weeks from the onset of pain to ischemic symptoms have been reported, although typically the delay is less than 24 hours.

The root lesion in VAD is an intimal tear or an expanding hematoma in the vessel wall. Subsequent neurologic deficits arise either because of ischemia from occlusion or because of distal artery to artery thromboembolism. The etiology of VAD may be either traumatic or spontaneous. The vertebral arteries are sensitive to injury from head or neck torsion because of their anatomy. They are most mobile at the level of C1 and C2 as they leave the transverse foramina and enter the intracranial cavity [2]. In most spontaneous cases, the cause is obscure but there is often a history of a minor precipitating event. In this case, the only notable event was the patient's air travel 4 weeks before presentation. Predisposing factors to dissection involve a combination of both genetic and environmental influences. They include heritable connective tissue diseases such as Marfan syndrome, Ehlers-Danlos syndrome type 4 and fibromuscular dysplasia.

Spinal manifestations of VAD are a rare clinical manifestation. Crum reported only 15 cases in his literature review [3]. The Brown-Séquard syndrome refers to the constellation of ipsilateral signs of posterior column and pyramidal tract dysfunction, with contralateral loss of pain and temperature due to involvement of the lateral spinothalamic tract. The syndrome commonly results from trauma, myelitis or cord hemisection. The clinical presentation of a Brown-Séquard syndrome with VAD as an underlying etiology has been reported only once before in the literature [4].

Advances in imaging techniques have allowed a greater recognition of VAD. Combined MR imaging and MR angiography examination is generally considered to be the best modality for initial evaluation of suspected VAD [5]. The resolution of MR angiography approaches that of conventional angiography, and it permits an evaluation of all the major arteries and the brain parenchyma itself. It has the added advantages of direct visualization of the intramural hematoma, indicated by abnormal signal intensity within the vessel wall. Typical findings are a periarterial rim of hematoma initially hyperintense on the T1 image and later on the T2 image, surrounded by either a narrow or sometimes normal eccentric signal void [6]. This hyperintensity usually persists for a number of months on MR imaging.

In the acute phase of brainstem or spinal cord ischemia, VAD should be actively sought out. Anticoagulation is typically employed for 3 to 6 months to prevent embolic consequences unless contraindications such as intracranial aneurysm or intradural dissection exist. Unfortunately, no randomized control trials have yet been conducted, nor is there evidence in related conditions such as carotid artery dissection [7]. Endovascular therapy with balloon angioplasty and stent insertion has shown some promise in short-term trials with carotid dissection. However, the long-term results are unknown and most dissections seem to heal spontaneously by themselves [8]. Most patients do well after a dissection, with one study demonstrating complete or very good recoveries in 88% of patients [9].

This case demonstrates the importance of considering arterial dissection in any relatively young patient with neck pain or stroke-like symptoms. Missing a diagnosis of VAD could have serious consequences, including intracranial dissection leading to subarachnoid hemorrhage and extending posterior infarction.

## Conclusion

We report an unusual presentation of a relatively uncommon condition. The case emphasizes the importance of considering dissection early on in any young patient with neck pain or stroke-like symptoms. Combined MR imaging and MR angiography is the best modality for initial evaluation, and with anticoagulation most patients do well.

## Abbreviations

C*n*: cervical vertebra *n*; CSF: cerebrospinal fluid; MR: magnetic resonance; MRC: Medical Researh Council; VAD: vertebral artery dissection.

## Consent

Written informed consent was obtained from the patient for publication of this case report and any accompanying images. A copy of the written consent is available for review by the Editor-in-Chief of this journal.

## Competing interests

The authors declare that they have no competing interests.

## Authors' contributions

SM acquired the patient records and data. The manuscript was written by SM and EM, and edited by DK. The manuscript was approved by all three authors before submission.

## References

[B1] Schievink W Spontaneous dissection of the carotid and vertebral arteries N Engl J Med 2001 344 898 906 10.1056/NEJM200103223441206 11259724

[B2] Thanvi B Munshi S Dawson S Robinson T Carotid and vertebral artery dissection syndromes Postgrad Med J 2005 81 383 388 10.1136/pgmj.2003.016774 15937204 PMC1743284

[B3] Crum B Mokri B Fulgham J Spinal manifestations of vertebral artery dissection Neurology 2000 55 304 306 10908913 10.1212/wnl.55.2.304

[B4] Goldsmith P Rowe D Jager R Kapoor R Focal vertebral artery dissection causing Brown-Séquard's syndrome J Neurol Neurosurg Psychiatry 1998 64 415 416 10.1136/jnnp.64.3.415 9527173 PMC2169977

[B5] Auer A Felber S Schmidauer C Waldenberger P Aichner F Magnetic resonance angiographic and clinical features of extracranial vertebral artery dissection J Neurol Neurosurg Psychiatry 1998 64 474 481 10.1136/jnnp.64.4.474 9576538 PMC2170029

[B6] Gelbert F Assouline E Hodes J Reizine D Woimant F George B Hagueneau M Merland J MRI in spontaneous dissection of vertebral and carotid arteries. 15 cases studied at 0.5 tesla Neuroradiology 1991 33 111 113 10.1007/BF00588245 2046892

[B7] Lyrer P Engelter S Antithrombotic drugs for carotid artery dissection Cochrane Database Syst Rev 2003 3 CD000255 12917890 10.1002/14651858.CD000255

[B8] Djouhri H Guillon B Brunereau L Levy C Bousson V Biousse V Arrive L Tubiana J MR angiography for the long-term follow-up of dissecting aneurysms of the extracranial internal carotid artery AJR Am J Roentgenol 2000 174 1137 1140 10749266 10.2214/ajr.174.4.1741137

[B9] Mokri B Houser O Sandok B Piepgras D Spontaneous dissections of the vertebral arteries Neurology 1988 38 880 885 3368069 10.1212/wnl.38.6.880

